# Defining ideal anterior hairline contour and hair-bearing proportion via a morphometric analysis in Chinese women

**DOI:** 10.3389/fsurg.2026.1946150

**Published:** 2026-09-09

**Authors:** Yiyi Zhang, Dadong Tang, Lanzhi Zhang, Jingping Wu, Fengrui Cheng, Hongbin Cheng

**Affiliations:** 1 School of Clinical Medicine, Chengdu University of Traditional Chinese Medicine, Chengdu, Sichuan, China; 2 Department of Cosmetology, Hospital of Chengdu University of Traditional Chinese Medicine, Chengdu, Sichuan, China; 3 Department of Dermatology, Hospital of Chengdu University of Traditional Chinese Medicine, Chengdu, Sichuan, China

**Keywords:** hair growth, hair treatment, hair-bearing proportion, hairline contour, morphometric analysis, statistics

## Abstract

**Objective:**

This study aimed to determine the ideal morphology of the anterior hairline contour and hair-bearing proportion in Chinese females, thereby improving preoperative planning protocols and enhancing clinical efficacy in both surgical hair transplantation and non-surgical camouflage interventions.

**Methods:**

Ninety participants completed a questionnaire-based study using digitally modified images of a female model. First, participants were randomly recruited to rate digitally altered images to investigate the most aesthetically preferred hairline contours. Five hairline contours (round, M-shaped, triangular, rectangular, and bell-shaped) were assessed across five dimensions: overall impression, naturalness, attractiveness, youthfulness, and agreeableness (on a 0–10 scale). Subsequently, respondents were asked to adjust the anterior hair-bearing proportion in the image until they reached their highest level of satisfaction. The height ratio (HR) and width ratio (WR) were calculated to quantify the hair bearing proportion.

**Results:**

Round, triangular, and M-shaped hairline contours were rated significantly higher than rectangular and bell-shaped contours in terms of overall appearance, naturalness, attractiveness, youthfulness, and agreeableness (all *p* < 0.001). The most preferred hair-bearing proportion corresponded to an HR of 0.505(IQR 0.455–0.584) and a WR of 0.373(IQR 0.356–0.387).

**Conclusion:**

Participants demonstrated a clear preference for round anterior hairline contours, with triangular and M-shaped contours also considered aesthetically favorable. An HR of approximately 0.505 and a WR of 0.373 represent the optimal anterior hair-bearing proportions. These population-specific findings provide quantitative evidence to support individualized planning for hair transplantation and nonsurgical aesthetic interventions.

## Introduction

The frontal hairline serves as a key aesthetic element in framing the face and defining the forehead contours. Receding, thinning, or disproportionate hairlines compromise facial symmetry and disrupt harmonious facial proportions (1). Hair loss can diminish femininity and attractiveness in women, contribute to an aged appearance, and ultimately result in psychological distress (2, 3). Therefore, techniques for optimizing the anterior hairline contour and hair-bearing proportion, such as hair transplantation and camouflage, are gaining widespread clinical adoption among women. An increasing number of studies have explored ideal hairline patterns for women to refine evidence-based strategies for hair restoration and aesthetic enhancement in this population. Naturally occurring female hairline patterns, including hairline contour shapes (4–6), hair growth direction (4) and measurement values of the infra-temporal portion of the forehead (6–8) have been discussed in previous studies. Several studies have identified ideal hairline contours across different cultures, indicating that an M-shaped hairline is predominantly favored in South Korea (9) and Spain (10), whereas a round hairline is preferred in Turkey (5). However, there is still a relatively limited understanding of aesthetic preferences regarding hairline contour among Chinese women.

The anterior hair-bearing region refers to the frontal and temporal portions of the scalp. The anterior hair-bearing proportion was defined as a normalized dimensionless index reflecting the relative extent of the hair-bearing region with respect to the forehead, calculated using linear measurements based on predefined anatomical landmarks. An adequate anterior hair-bearing proportion contributes to facial contour harmonization and a youthful appearance. In contrast, an insufficient anterior hair-bearing proportion may impart an aged and fatigued appearance, whereas an excessive proportion may compromise the naturalness and aesthetic authenticity.

This study investigated public preferences for female anterior hairline contours and hair-bearing proportions, which may help inform the optimization of reconstructive surgical planning and development of camouflage cosmetics tailored for Asian women.

## Materials and methods

Between November 2023 and February 2024, a total of 100 individuals were randomly recruited for this study at the Beauty Clinic of the Affiliated Hospital of Chengdu University of Traditional Chinese Medicine. Participants were informed of the study through posters and direct invitation during their clinic visits, and completed the digital questionnaire on site. To minimize social desirability bias, all participants were blinded to the specific research hypothesis and were only informed that this was a general survey on facial perception. Ultimately, 90 participants provided complete valid responses. The target sample size was determined based on projected attrition and established conventions in perceptual rating research. We initially recruited 100 participants, accounting for an expected 10%–20% rate of incomplete or invalid responses, to ensure a minimum of 80 valid raters for the final analysis. Previous aesthetic perception studies employing similar within-subjects designs and continuous rating scales have demonstrated that a sample of 75–90 participants is sufficient to detect medium to large effect sizes (partial *η*^2^ = 0.13–0.55) (11). Besides, a *post-hoc* power analysis was performed using G*Power 3.1 for the primary repeated-measures ANOVA. With a two-tailed *α* level of 0.05, 5 within-subject levels, a conservative estimated correlation of 0.5 between repeated measures, and a conventional medium effect size (*f* = 0.25), the analysis yielded a statistical power greater than 0.95. All participants voluntarily completed the questionnaires. Respondents were required to provide demographic information anonymously, including sex, age, occupation and education level. This study adhered to the principles of the Declaration of Helsinki, and was approved by the Medical Ethics Committee of the Affiliated Hospital of Chengdu University of Traditional Chinese Medicine (Reference Number: 2025KL-046). Written informed consent for publication was obtained from all participants included in the study.

## Study design

**Figure 1:**
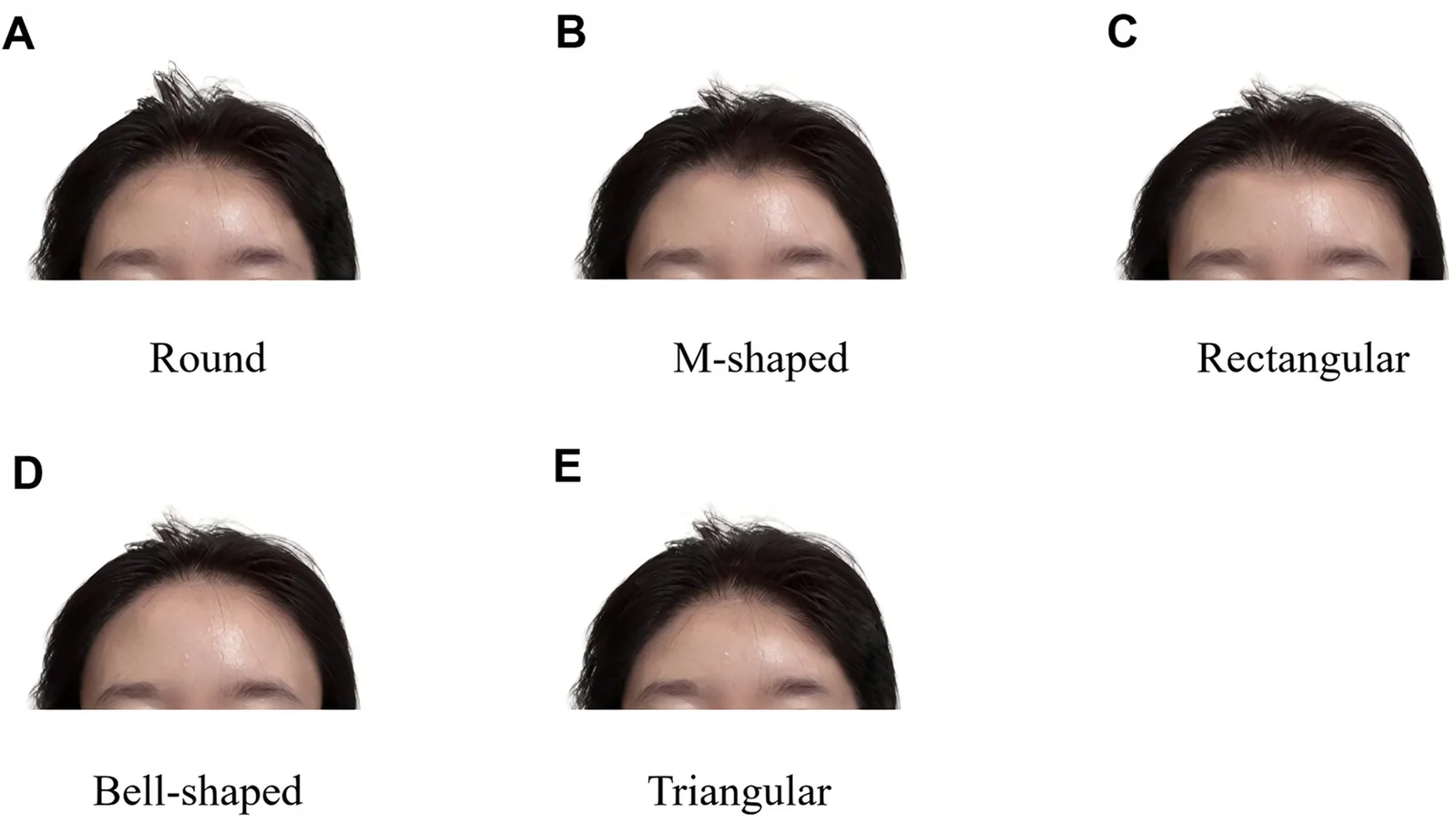
Images of classification of female anterior hairline contour. **(A)** Round type: features a smooth concave hairline contour with no distinct frontotemporal recession; **(B)** M-shaped type: presents marked frontotemporal recession, an upward-displaced lateral frontal hairline, and a frontotemporal angle generally less than 90°; **(C)** Rectangular type: demonstrates a horizontally aligned frontal hair margin, with mild frontotemporal recession and a frontotemporal angle of 90° or larger; **(D)** Bell-shaped type: maintains a normal forehead width, while the midpoint of the frontal hairline is approximately 2 cm higher than the population average; **(E)** Triangular type: shows a nearly straight, descending hairline from the central forehead to the temporal region, with abundant vellus hairs distributed along the hairline edge.

Images were obtained from a young female participant with no history of alopecia, scalp surgery, or congenital craniofacial anomalies. Written informed consent for publication was obtained from the participants prior to image collection. Images were edited using Photoshop (Version CC2020) and Meitu (version 4.0), with only the hairline adjusted and all other facial features and image parameters held constant. First, in reference to Suzan et al. (5, 12) the hairline in the image was modified into several distinct shapes, including round, M-shaped, rectangular, bell-shaped, and triangular. The classification of female hairline contours is shown in Figure 1. All evaluation tasks were performed on identical 10.5-inch tablets (1920 × 1200 pixels) at a fixed viewing distance of 30 cm, with the image zoom function disabled to maintain consistent visual perception across participants. To accurately assess the aesthetic preferences of Chinese individuals regarding frontal hairline patterns, participants were instructed to rate the images across five dimensions: overall impression, naturalness, attractiveness, youthfulness, and agreeableness using a scale ranging from 0 to 10. A score of 0 indicated complete nonsatisfaction, while a score of 10 indicated complete satisfaction. Subsequently, participants were asked to adjust the anterior hair-bearing region in the images by Meitu until they reached their highest level of satisfaction.

The hair-bearing proportion was quantified using two proportional indices: height ratio (HR) and width ratio (WR). The HR was defined as the ratio of the hair-bearing region height to the forehead height, while WR was defined as the ratio of the hair-bearing region width to the forehead width.

The forehead height was defined as the vertical linear distance between the trichion (Tr) and glabella (G) points (13). The forehead width was defined as the linear distance between the bilateral intersections of the hairline and a horizontal reference line drawn through the lateral brow arch points (LBA).

**Figure 2:**
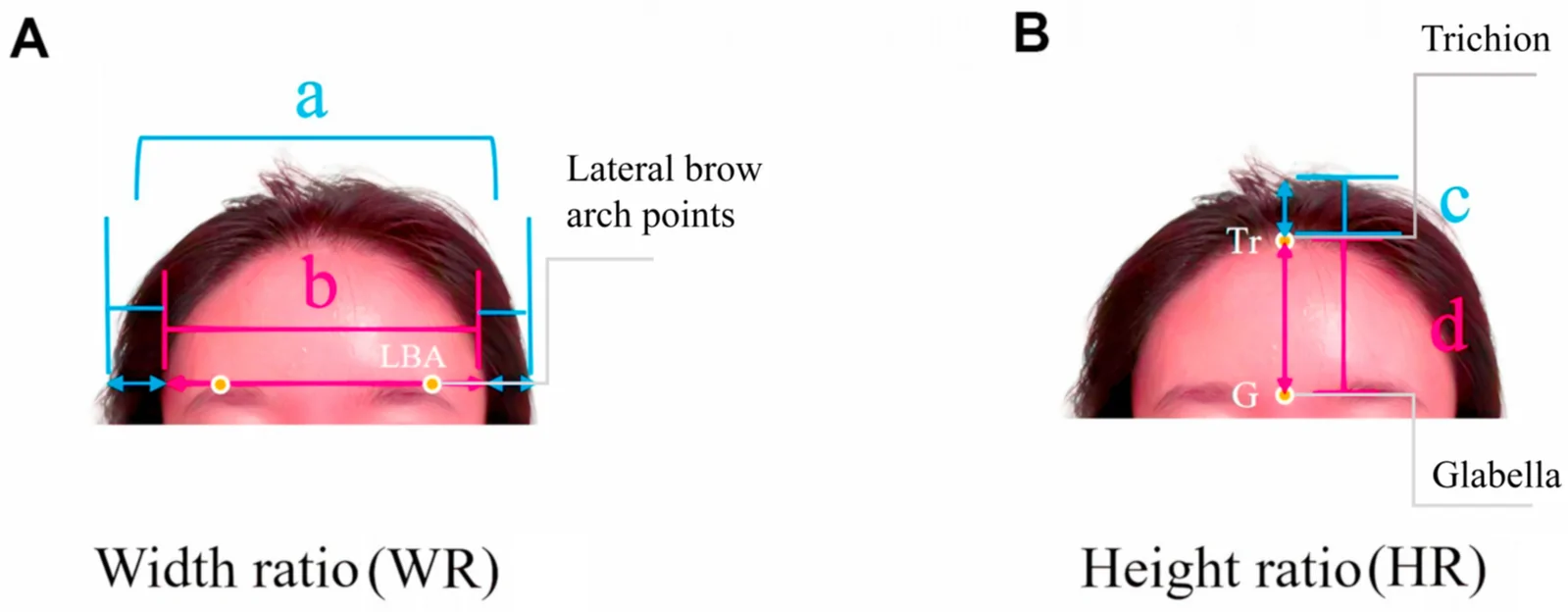
Definition and measurement of hair-bearing proportion indices. **(A)** Width ratio (WR) = a/b, where a = width of the hair-bearing region, and b = forehead width. Forehead width **(b)** was measured as the horizontal distance between the bilateral intersections of the hairline with a reference line passing through the lateral brow arch points **(LBA)**. The hair-bearing region width **(a)** was measured as the transverse distance between its bilateral lateral boundaries along the same reference line. **(B)** Height ratio (HR) = c/d, where c = height of the hair-bearing region, and d = forehead height. Forehead height **(d)** was measured as the vertical distance from the trichion **(Tr)** to the glabella **(G)** The hair-bearing region height **(c)** was measured as the vertical distance from the trichion **(Tr)** to the superior boundary of the anterior hair-bearing region.

The height of the hair-bearing region was defined as the vertical linear distance from the trichion (Tr) to the superior boundary of the anterior hair-bearing region. The width of the hair-bearing region was defined as the transverse linear distance between its bilateral lateral boundaries measured along the same horizontal reference line. The HR and WR measurements are shown in Figure 2.

## Statistical analysis

Statistical analyses were performed using the SPSS software (version 25.0; IBM Corp., Armonk, NY, USA). Categorical demographic variables are presented as frequencies and percentages. All subjective rating scores and preferred proportion values were tested for normality via the Shapiro–Wilk test.

Since the data deviated significantly from normal distribution, Friedman rank-sum test was used to examine differences in hairline contours preference. When the global test yielded a statistically significant result (*P* < 0.05), paired Wilcoxon signed-rank tests with Bonferroni correction were performed for all pairwise comparisons (*P* < 0.005). Subgroup analyses by sex and educational level were performed using the Scheirer–Ray–Hare test to examine the main effects and interaction effects between hairline contour and each demographic variable.

Shapiro–Wilk tests demonstrated that the preferred HR deviated significantly from normal distribution (*P* < 0.001), whereas the preferred WR approached approximate normality. To preserve consistency with the overarching nonparametric analytical framework applied to all hairline contour ratings, all continuous proportion outcomes are presented as medians with interquartile ranges (IQRs), and all subgroup comparisons stratified by sex and educational level were uniformly conducted using the Mann–Whitney U test. Spearman's rank correlation analysis was used to investigate correlation between the HR and WR.

## Results

### Demographic characteristics

A total of 90 valid participants were included in the final analysis. The mean age of the participants was 28.16 ± 4.91 years. In terms of professional background, 75 participants (83.3%) had no systematic academic or professional background in aesthetics, while 15 participants (16.7%) majored in aesthetics or received formal professional aesthetics training. The gender distribution was relatively balanced, with 46 males (51.1%) and 44 females (48.9%). Regarding educational attainment, 44 participants (48.9%) had an educational level of bachelor degree or below, and 46 participants (51.1%) held a master degree or above. Subgroup analyses were performed to examine variations in aesthetic preferences across population characteristics. Given that the study sample was predominantly concentrated in the 20–30 years age range, and the number of participants with a professional aesthetics background was relatively small with a markedly unbalanced group distribution— which would result in insufficient statistical power for reliable between-group comparisons—subgroup analyses were restricted to two variables: gender and educational attainment. Demographic characteristics of the participants is shown in Table 1.

**Table 1:** Demographic characteristics of the study participants.

Characteristic	Value
Age, years, mean ± SD	28.16 ± 4.91
Sex, *n* (%)	
Male	46 (51.1)
Female	44 (48.9)
Professional background, *n* (%)	
No formal aesthetic training	75 (83.3)
Formal aesthetic background	15 (16.7)
Educational level, *n* (%)	
Bachelor degree or below	44 (48.9)
Postgraduate degree or above	46 (51.1)

Continuous variables are presented as mean ± standard deviation; categorical variables are presented as *n* (%). Total sample size *N* = 90.

### Optimal anterior hairline contours

In terms of overall appearance, naturalness, attractiveness, youthfulness, and agreeableness (all *p* < 0.001), the round, triangular, and M-shaped anterior hairline contours were rated more favorably than the bell-shaped and rectangular contours. Among them, the round contour received the highest median score, followed by the triangular and M-shaped contours, while no statistically significant pairwise differences were found among the three after Bonferroni correction. In contrast, the rectangular contour received the lowest score, with the bell-shaped contour ranking second lowest.

**Figure 3:**
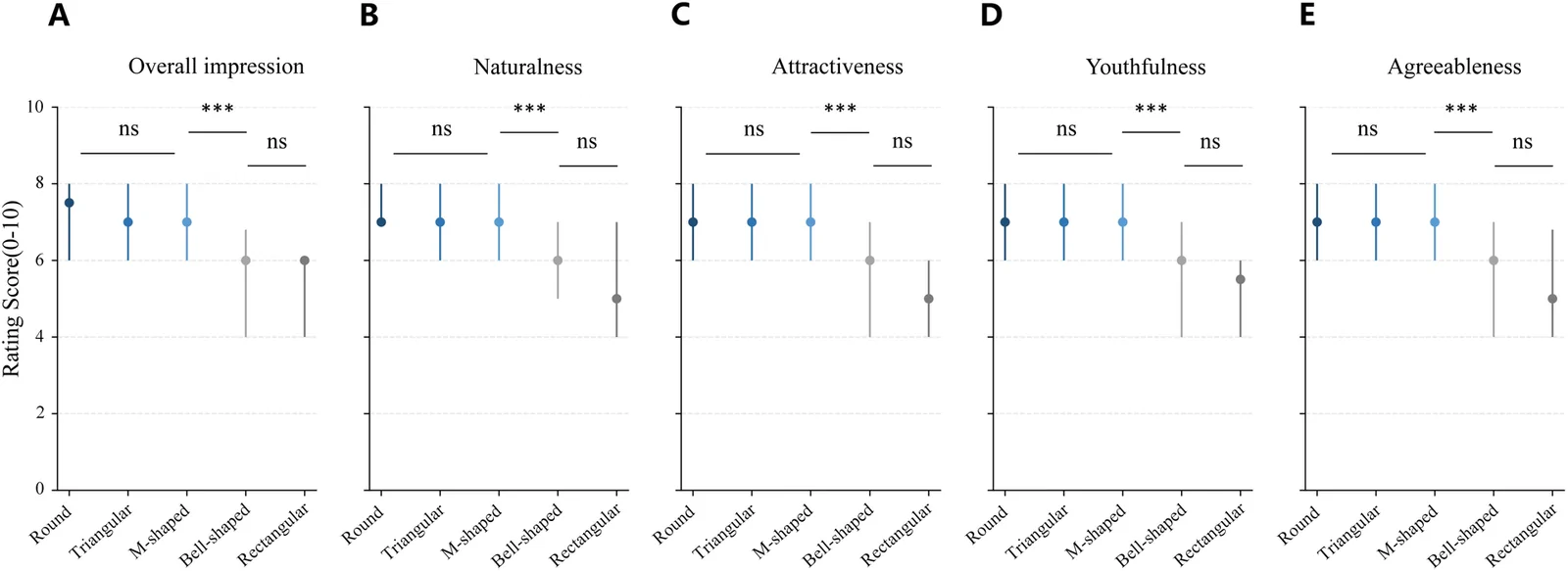
Aesthetic ratings of five hairline contours across five evaluation dimensions. Aesthetic scores of five hairline contours (Round, Triangular, M-shaped, Bell-shaped, Rectangular) were evaluated across five domains: **(A)** Overall impression, **(B)** Naturalness, **(C)** Attractiveness, **(D)** Youthfulness, and **(E)** Agreeableness. Statistical comparisons were performed using the Friedman test followed by *post-hoc* pairwise tests; ***P < 0.001; *ns*, no statistically significant difference.

Aesthetic ratings of five hairline contours across five evaluation dimensions is shown in Figure 3. Detailed score comparisons across the five contour shapes are available in the Supplementary Tables 1, 2.

### Ideal anterior hair-bearing proportion

**Figure 4:**
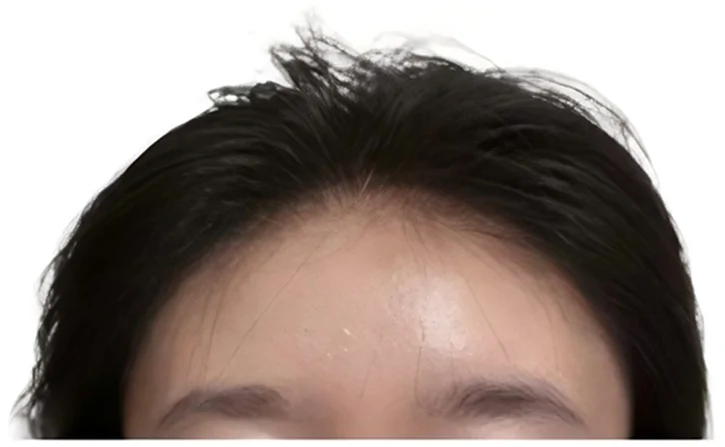
Representative image of the most preferred anterior hair-bearing proportion. The optimal hair-bearing ratios corresponding to the highest participant satisfaction are a mean height ratio (HR) of 0.505(IQR 0.455–0.584) and a mean width ratio (WR) of 0.373(IQR 0.356–0.387), derived from participant-guided interactive image adjustment.

Based on 90 valid samples, the median preferred HR was 0.505, IQR 0.455–0.584, and the median preferred WR was 0.373, IQR 0.356–0.387. These values represent the proportions at which participants reported the highest level of satisfaction following interactive adjustment of the hair-bearing region. Spearman's rank correlation analysis revealed a weak but significant positive correlation between the HR and WR (*ρ* = 0.363, *P* = 0.0004). Preferred hair-bearing proportions are presented in the Supplementary Table 4. A representative image corresponding to the preferred anterior hair-bearing proportion is shown in Figure 4.

### Males vs. females

For all five rating dimensions, both male and female participants consistently rated round, triangular, and M-shaped hairlines significantly higher than bell-shaped and rectangular hairlines (all *p* < 0.001). No significant interaction effect between sex and hairline contour was detected (all *p* > 0.05), indicating highly consistent preference patterns across sexes.

The median preferred HR was 0.495 (IQR: 0.450–0.587) in males and 0.515 (IQR: 0.455–0.575) in females. The median preferred WR was 0.376 (IQR: 0.359–0.392) in males and 0.369 (IQR: 0.353–0.381) in females. No significant sex differences were found for either HR (*P* = 0.608) or WR (*P* = 0.264). Subgroup analyses by sex are provided in the Supplementary Tables 3, 4.

### Bachelor degree or below vs. master degree or above

Across all five dimensions, participants with different educational levels showed an identical preference hierarchy: round, triangular, and M-shaped hairlines received higher ratings than bell-shaped and rectangular hairlines (all *p* < 0.001). No significant interaction effect between educational level and hairline contour was observed (all *p* > 0.05).

The median preferred HR was 0.486 (IQR: 0.450–0.559) in participants with a bachelor's degree or below, and 0.520 (IQR: 0.472–0.599) in those with a postgraduate degree. The median preferred WR was 0.375 (IQR: 0.360–0.388) and 0.372 (IQR: 0.353–0.387), respectively. No significant educational differences were found for either HR (*P* = 0.116) or WR (*P* = 0.583). Subgroup analyses by education level are shown in the Supplementary Tables 3, 4.

## Discussion

The increasing prevalence of pattern hair loss in women has led to a growing demand for therapeutic and cosmetic interventions aimed at restoring or enhancing the anterior hairline contour (14). To optimize treatment outcomes, research on the aesthetics of female hairlines has gained increasing attention. Previous studies have described frontal hairline contours across different populations; however, most have focused on naturally occurring patterns rather than on subjective aesthetic preferences. Notably, the most prevalent hairline shape is not necessarily the most aesthetically preferred (15).

In the present study, subjective aesthetic evaluations were quantitatively assessed to identify preferred anterior hairline contours in a Chinese population. Based on the established classifications (4, 5), five contour types were analyzed: round, bell-shaped, M-shaped, rectangular, and triangular. Prior studies from South Korea and Spain reported that M-shaped hairlines were the most attractive, followed by rectangular and round contours (9, 10); M-shaped hairlines were considered the most attractive, followed by rectangular and round contours. However, studies in Turkey have suggested that round hairlines are perceived as more youthful, healthy, and feminine (5).

In this study, the round hairline contour received the highest scores across all evaluated dimensions, followed by the triangular and M-shaped contours; however, pairwise comparisons revealed no statistically significant differences among these three shapes (all *P* > 0.05). In contrast, the bell-shaped and rectangular contours were consistently rated less favorably.

Our findings are consistent with those reported in Turkey but differ from those of studies conducted in Korea and Spain. The round hairline contour is widely regarded as the typical and optimal pattern in women (5, 6). Its smooth, continuous curvature enhances facial framing and is associated with increased perceptions of femininity and agreeableness (16, 17). Accordingly, the round contour received the highest mean rating across all dimensions evaluated in this study. Differences in preferences for M-shaped hairlines across populations can be explained by both cultural aesthetic orientations and anthropological anatomical foundations. Despite similarly round facial contours in Chinese and Korean East Asian populations, Korean aesthetic preferences align more closely with popular trends and favor hairline forms with subtle structural details, as the M-shaped contour can visually reduce perceived facial width; in contrast, Chinese participants place greater emphasis on overall facial harmony, and a round hairline better echoes the traditional aesthetic preference for soft, rounded facial features. For the Spanish Caucasian population, the high acceptance of the M-shaped hairline is rooted in natural physiological traits: up to 94.17% of healthy Spanish women are naturally born with a widow's peak, and the undulating M-shaped contour transitions naturally with the prominent brow ridges and frontal tuberosities of the Caucasian facial skeleton, delivering higher anatomical coordination and aesthetic compatibility.

Feminized foreheads are generally characterized by reduced vertical height and minimal temporal exposure (16, 18). In line with this, the triangular contour was also positively evaluated, possibly because of reduced forehead exposure and a more youthful appearance. By contrast, bell-shaped and rectangular contours, which are associated with increased forehead height and greater temporal exposure, were consistently perceived as less aesthetically pleasing.

However, the aesthetic perception of M-shaped hairlines remains controversial. While some studies associate this pattern with masculinity (17, 19), others have reported favorable aesthetic ratings (2, 10). In the present study, the M-shaped contour received intermediate scores, suggesting a variability in individual preferences. As previously discussed, preference for round hairlines consistent with the traditional aesthetic emphasis on soft facial harmony. Nevertheless, under the influence of Korean popular culture and Western aesthetic trends, and given evidence that mild M-shaped hairlines (widow's peaks) are not linked to androgen levels or masculine features (10), a proportion of participants view this contour positively, which accounts for its intermediate overall rating.

In addition to the contour shape, the anterior hair-bearing proportion is a critical factor in the design of female hair transplantation and camouflage strategies. This proportion, defined as a normalized index reflecting the relative extent of the hair-bearing region to the forehead, decreases with age because of hair thinning and frontal hairline recession (20, 21). Therefore, the restoration of an appropriate hair-bearing proportion is a key objective in clinical practice. However, evidence regarding the ideal hair-bearing proportions remains limited. In the present study, an HR of 0.505 and a WR of 0.373 received the highest aesthetic ratings among the participants. Numerically, the height ratio of 0.505, close to the 0.5 midpoint, aligns with the traditional Chinese aesthetic emphasis on balance and moderation; this near-even proportion avoids the visual imbalance of excessively high or low hairlines and creates a well-proportioned forehead appearance. The width ratio of 0.373 is also close to the complement of the golden ratio (0.382), a classical proportional reference commonly discussed in facial aesthetic studies, which may partially account for the harmonious horizontal contour perceived by participants. To explore the relationship between preferred HR and WR, we performed Spearman's rank correlation analysis, which showed a weak but significant positive correlation (*ρ* = 0.363, *P* = 0.0004), indicating the two proportions are not fully independent. This interdependence indicates that HR and WR should be adjusted synergistically rather than in isolation in clinical hairline design, to better achieve an overall harmonious aesthetic outcome. Collectively, these optimal ratios integrate universal visual perceptual rules and culturally rooted aesthetic preferences, explaining the highest satisfaction with corresponding hair-bearing contours.

Subgroup analyses demonstrated minimal differences in aesthetic preferences across gender and educational level, indicating that these findings are relatively robust and not significantly influenced by demographic factors.

Beyond statistical significance, these findings hold direct clinical value for hairline aesthetic design. The higher-rated round and triangular hairlines better align with mainstream public aesthetic preferences, while the M-shaped contour provides a personalized option. The optimal HR and WR derived from this Chinese cohort offer a localized reference, reducing reliance on data from other ethnic populations. The optimal HR and WR identified in this Chinese cohort provide a reference for surgical hairline design in this population. The weak positive correlation between the two ratios further supports synergistic adjustment of height and width to maintain proportional harmony. Given the modest score gaps among top-rated contours, these findings are design references rather than rigid standards, with individualized planning remaining the core clinical principle.

This study had several limitations. Firstly, the participants were recruited from the cosmetic department of a tertiary hospital and were predominantly young, and only a single facial model was used to generate the stimulus images—both of which may limit the generalizability of our findings. Future studies with larger, more diverse populations across multiple centers are warranted to validate and extend our results. Secondly, digitally modified images may not fully capture real-world perceptual experiences; therefore, clinical practice necessitates individualized adjustments tailored to each patient's unique facial anatomy and aesthetic characteristics. Thirdly, despite blinding participants to the study hypothesis, social desirability bias may persist in aesthetic evaluations, as participants might respond according to perceived social norms rather than genuine preferences. Finally, the self-developed proportion adjustment task lacks standardized reliability and validity validation, and its reproducibility requires further verification. Nevertheless, to our knowledge, this is the first study to evaluate both the anterior hairline contour and hair-bearing proportion in a Chinese population, providing valuable evidence to guide the design of surgical and nonsurgical aesthetic interventions.

## Conclusion

This study provides the first systematic report on optimal anterior hairline contours and hair-bearing proportions in Chinese females. Round, triangular and M-shaped hairline contours achieved favorable aesthetic ratings across all evaluated dimensions, with the optimal height ratio (HR) and width ratio (WR) identified as 0.505 and 0.373, respectively. These population-specific results serve as a practical evidence-based reference for clinical decision-making in hair restoration surgery and nonsurgical hairline camouflage strategies. Further multicenter studies with larger, more diverse populations will help validate these findings and enable their broader clinical application.

## Data Availability

The raw data supporting the conclusions of this article will be made available by the authors, without undue reservation.
